# Sexualized Behavior Among Adolescents Who Sexually Offended

**DOI:** 10.1007/s10508-022-02345-0

**Published:** 2022-09-28

**Authors:** Chiara Krause, Steffen Barra, Markus A. Landolt, Cornelia Bessler, Marcel Aebi

**Affiliations:** 1 Department of Forensic Psychiatry, Centre for Child and Youth Forensic Therapy, University Hospital of Psychiatry, Neptunstrasse 60, 8032 Zurich, Switzerland; 2 Institute for Forensic Psychology and Psychiatry, Saarland University Hospital, Homburg, Germany; 3 Division of Child and Adolescent Health Psychology, Department of Psychology, University of Zurich, Zurich, Switzerland; 4 Department of Psychosomatics and Psychiatry, University Children’s Hospital Zurich, Zurich, Switzerland; 5 Department of Justice and Home Affairs, Canton of Zurich, Research and Development, Corrections and Rehabilitation, Zurich, Switzerland; 6 Department of Child and Adolescent Psychiatry and Psychotherapy, University Hospital of Psychiatry, Zurich, Switzerland

**Keywords:** Sexualized behavior, Sexual behavior problems, Juvenile sexual offending, Sexual recidivism

## Abstract

Early or excessive sexualized behaviors and preoccupations with sexuality (SB) exhibited by juveniles who have sexually offended (JSO) are considered risk factors for sexual recidivism. However, research into SB among JSO is scarce. The present study retrospectively examined prevalence rates and patterns of SB among JSO prior to sexual offending and their relation to psychopathology and sexual recidivism. We systematically assessed information from psychiatric and psychological expert reports in case files of 230 JSO aged 12–18 years (*M* = 14.46, SD = 1.49) from a population sample of JSO with contact sexual offenses. A total of 93 (40.4%) JSO exhibited SB prior to the index sexual offense. Latent class analysis revealed three SB profiles: (1) “low/no SB” (*n* = 188), (2) “preoccupied SB” (preoccupation with sexuality, e.g., early pornography consumption, excessive masturbation; *n* = 29), and (3) “dysregulated SB” (exhibiting inappropriate sexualized behaviors toward others, e.g., sexualized speech, touching others inappropriately; *n* = 13). The preoccupied SB and the dysregulated SB groups showed higher prevalence of psychiatric disorders than the low/no SB. However, none of the JSO of the preoccupied SB or dysregulated SB groups reoffended sexually within 365 days after conviction for the sexual index offense (low/no SB: 12.8%). Overall, our findings do not support a general notion of the presence of SB as an indicator of high risk for persistent sexual offending among JSO. Instead, JSO with SB appear particularly burdened regarding a range of psychiatric disorders that should be treated accordingly.

## Introduction

Recent decades have seen increasing concern about the impact that extensively sexualized media has on childhood development (e.g., Albury, 2013; Hill, 2011). Nowadays, children are routinely exposed to sexualized and/or sexually explicit content by both traditional sources (e.g., TV, billboards, magazines) and new media (e.g., internet, apps, chats) (e.g., Albury, 2013; Efrati, 2020). Research indicates that exposure to sexualized content contributes to premature and problematic sexual preoccupations, interests, and behaviors, including coercive sexual behaviors among youth (DeLago et al., 2020; Dillard et al., 2019; Efrati, 2020; Hill, 2011; Lillie, 2017; Ybarra et al., 2011). However, differentiating non-normative from normative sexual development in childhood and adolescence is challenging, and definitions vary (e.g., Chaffin et al., 2008; Elkovitch et al., 2009; Russell, 2020). In fact, even normative sexual development across childhood is not well understood (Elkovitch et al., 2009; Lamb & Plocha, 2017). Research is limited due to difficulties in measuring childhood sexual behaviors, such as the taboo of reporting children’s sexual behaviors, cultural differences, and the change of norms over time.

In a consensus definition, normative sexual behavior by children has been described as involving sex play and exploration that occurs spontaneously and infrequently and is both mutual and noncoercive (Chaffin et al., 2008). The term “sexual” used in this context does not necessarily imply that the motivation of the behavior is sexual (Chaffin et al., 2008). Available research suggests childhood sexual behaviors are common and children show a wide range of individual and shared sexual behaviors across childhood (Friedrich et al., 1991; Sandfort & Cohen-Kettenis, 2000). For an in-depth discussion of childhood sexual development see DeLamater and Friedrich (2002). Details on normative vs. concerning sexual behavior in childhood for different ages can for example be found in Wurtele and Kenny (2011).

### Sexualized Behavior

Substantial research effort into non-normative sexual behavior in childhood has been made by Friedrich and colleagues, including the development of an instrument to measure problematic childhood sexual behavior (Friedrich et al., 1992, 2003). Generally, problematic or non-normative childhood sexual behavior is understood as behavior that is not considered developmentally adequate, too frequent (e.g., to the exclusion of other activities or leading to social isolation), considered unacceptable by society, which causes harm or other negative consequences to self or others, or which does not respond to parenting (Chaffin et al., 2008; Elkovitch et al., 2009; Lussier et al., 2018). Definitions of non-normative childhood sexual development vary but commonly include inappropriate, early or excessive *sexualized behaviors/preoccupations* (SB) (e.g., early/excessive masturbation or touching others inappropriately; Chaffin et al., 2008; Elkovitch et al., 2009), and—regarding later childhood—deviant sexual interests, fantasies, and arousal (e.g., Davis & Knight, 2019; Prentky & Righthand, 2003; Worling, 2012). In the present study, we consider SB separately from sexual deviance/deviant sexual interests.

Available research suggests that children with SB tend to present high rates of psychopathology, externalizing problems (Ensink et al., 2018; Letourneau et al., 2004; Tarren-Sweeney, 2008) and adverse childhood experiences are especially common (e.g., Lussier et al., 2019; Silovsky & Niec, 2002; Szanto et al., 2012). Particular attention has been directed toward the link between SB and exposure to sexual abuse (Elkovitch et al., 2009; Friedrich et al., 2003). However, a history of sexual abuse appears not to be a necessary factor for SB (Allen, 2017; Ensink et al., 2018; Lévesque et al., 2010; Silovsky & Niec, 2002; Szanto et al., 2012), but is rather one of multiple possible factors contributing to the etiology of SB (for a review, see Elkovitch et al., 2009). A concern with children who exhibit SB is whether SB will progressively evolve into sexually coercive/delinquent behaviors over time (e.g., Carpentier et al., 2006; Leach et al., 2016; Swisher et al., 2008). Longitudinal research suggests that this is very rarely the case (e.g., 2% in Carpentier et al., 2006; Letourneau et al., 2008) and that SB respond well to treatment (Barry & Harris, 2019; Carpentier et al., 2006; Letourneau et al., 2004; Silovsky et al., 2007).

### Sexualized Behavior and Juvenile Sexual Offending

Literature on juveniles who sexually offended (JSO) suggests that atypical sexual development (e.g., exposure to pornography or sexual preoccupation, Seto & Lalumiere, 2010) and prior sexually transgressive behaviors (e.g., Burton, 2000) are characteristic for JSO. The role of SB as risk factors for persistent sexual offending by JSO is not well understood. While SB are considered relevant for sexual offending, research focusing specifically on SB is lacking, with available research focusing on deviant sexual interests, fantasies, arousal, or a combination of SB and sexual deviance as risk factors (e.g., Clift et al., 2009; Dennison & Leclerc, 2011; Kenny et al., 2001; McCann & Lussier, 2008; Prentky et al., 2000; Worling, 2012). Consequently, the role of SB displayed by JSO independent of sexual deviance is not well understood as a risk factor for either general or sexual recidivism.

Most risk assessment instruments for JSO consider SB a risk factor for sexual recidivism. For example, the ERASOR item 2 (Worling & Curwen, 2001, coding form p. 2) codes “Obsessive sexual interests/Preoccupation with sexual thoughts” operationalized as unusually frequent sexual thoughts, comments, gestures, or behaviors, pornography use, sexual fantasy, or the excessive use of sexual behaviors/fantasies to cope with negative affect, anger, or problematic situations. Similarly, the J-SOAP-II (Prentky & Righthand, 2003), one of the most widely used risk assessment instruments for sexual recidivism among JSO, includes SB in the risk item “sexual drive/preoccupation,” specified as “evidence of an excessive amount of sexual activity (exceeding what might be considered normative for youths of that age) or excessive preoccupation with sexual urges or gratifying sexual needs. Evidence includes, but is not limited to, paraphilias (exposing, peeping, cross-dressing, fetishes, etc.); compulsive masturbation; chronic and compulsive use of pornography; frequent highly sexualized language and gestures; and indiscriminate sexual activity with different partners out of the context of any relationship.” (J-SOAP-II manual, Prentky & Righthand, 2003, p.15). However, the authors caution that this risk factor is not well understood: “The role of deviant sexual arousal, sexual drive, and sexual preoccupation as risk factors in juvenile sexual aggression, however, is quite unclear.” (Prentky et al., 2000, p. 84). Two decades on, a dearth of research remains into SB among JSO, and their relevance to the prediction of criminal recidivism remains largely an assumption.

In sum, while SB are considered relevant factors in sexual offending by adolescents, research is scarce and complicated by the use of varying definitions of SB and their overlap with sexual deviance. Consequently, no coherent picture of the role of SB in adolescent sexual offending behavior emerges. Therefore, the objectives of the present study were twofold. First, we aimed to explore patterns of SB exhibited by JSO prior to their sexual index offense based on descriptions in psychiatric and psychological expert reports in case files using latent-class analysis. Given previous research on the relevance of SB in some JSO (Prentky & Righthand, 2003; Prentky et al., 2000), we expected to find at least one group with no or low SB and one group with higher SB. Second, we aimed to compare possible subtypes of SB in JSO with regard to sexual victimization, psychiatric disorders, and criminal characteristics, including risk estimates and sexual and general recidivism. We assumed that JSO with SB would show higher rates of sexual victimization and psychiatric disorders than JSO with no/low SB, would score higher on the J-SOAP-II, and—as indicated by the J-SOAP-II manual—would show higher rates of sexual recidivism. Given the previous findings on SB and externalizing behavior problems (Ensink et al., 2018; Letourneau et al., 2004; Tarren-Sweeney, 2008) we further tested SB as predictor of violent and general recidivism.

## Method

### Sample

In total, data were available from case files of 687 JSO (*n* = 673, 98.0% boys, *n* = 14, 2.0% girls). To ensure sufficient details in the case files to code SB and psychiatric disorders and to comply with the restrictions on the risk assessment instrument (J-SOAP-II), we only included male JSO between 12 and 18 years of age who had been convicted of a contact sexual offense for whom a psychiatric or psychological assessment by a forensic expert was available in the case files. Accordingly, the final sample for the present study included case files of 230 JSO with a mean age of 14.46 (SD = 1.49) at the time of the index sexual offense. A total of 73 (31.7%) JSO were of foreign nationality, and 35 (15.2%) came from a family background with low SES.

### Procedure

This study is based on a population sample including all JSO convicted of a sexual offense between January 2007 and September 2014 in 14 German-speaking cantons (states) of Switzerland. Data was retrospectively extracted from the case files of juvenile justice authorities between February and December 2015 by an experienced forensic researcher, a Ph.D. student, and a research assistant from the University Hospital of Psychiatry Zurich. Coding was guided by a specifically developed documentation system based on an adaptation of the Forensic Psychiatric Documentation System (Nedopil et al., 1986). It was modified for juveniles and expanded to assess family background (including adverse childhood experiences), offense characteristics, and psychiatric diagnoses (see also Barra et al., 2017, 2018). For the present study, information on demographics, SB, psychiatric diagnoses, previous offenses, offense characteristics, and risk items of the J-SOAP-II were gathered from case files. Information on criminal recidivism was coded from case files and official databases. Case files of 30 JSO were randomly selected from the original sample and blindly rated by a second rater. Inter-rater agreement was calculated subsequently (nominal variables: Cohen’s κ; metric variables: intra-class correlation coefficient [ICC] two-way random model, single measure, absolute agreement).

All study procedures were approved by the ethics committees of the Canton of Zurich, Central Switzerland, and Northwest Switzerland (lead ethics committee: Zurich, EC-No. 2010-0483) and by the juvenile justice institutions concerned. No informed consent from JSO or parents/legal guardians was necessary for retrospective file access or for official data on re-offenses.

### Measures

#### Demographic Information

Foreign nationality was defined as not holding Swiss citizenship. We classified parental employment according to the International Standard Classification of Occupations (ISCO-08) norms published by the International Labor Organization (2012). We considered the socio-economic status (SES) as low if both parents were either unskilled workers (ISCO-08 code 9) or unemployed or if either was the case for one of the parents and information was missing on the employment of the other parent. For participants charged with multiple sexual offenses, age at the first occurrence of the index offense was used as age at index offense.

#### Sexualized Behaviors Exhibited Before the Index Sexual Offense

Coding of SB was based on the definition used in the J-SOAP-II, item 7 sexual drive and preoccupation: “evidence of an excessive amount of sexual activity (exceeding what might be considered normative for youths of that age) or excessive preoccupation with sexual urges or gratifying sexual needs.” (Prentky & Righthand, 2003, p.15). We considered the following SB: (1) indication of excessive sexual thoughts (multiple times per day), (2) indication of inappropriate touching of others, (3) indication of undressing or showing genitalia inappropriately, (4) indication of masturbation in the presence of others, (5) indication of early masturbation (before age 12), (6) indication of excessive masturbation (multiple times per day), (7) indication of early use of pornography (before age 12), and (8) indication of excessive pornography consumption (multiple times per day). The age of 12 years as cut-off criterion for the items 5 and 7 was used in line with previous research (Friedrich et al., 1998). In line with item 7 of the J-SOAP-II, no age range was specified for the SB (except for early masturbation and early pornography). However, to ensure SB were not confounded with the index sexual offense, only SB prior to the index sexual offense were considered independent of whether these behaviors led to criminal charges. Information on these SB was coded from psychiatric and psychological expert reports in the case files. SB indicators were dichotomously coded as present or absent, and the age range was taken from the files. Kappa showed substantial overall agreement between raters (*κ* = 0.77) ranging from moderate to almost perfect agreement for the dichotomous SB items (*κ* = 0.58–1.00) (Landis & Koch, 1977). Since a wide age span was included (0–17.99 years), judgement for the age range was considered congruent if the age range coded was equal or the deviation did not exceed one year. There was substantial agreement regarding the age ranges during which the SB were exhibited (*κ* = 0.72). For the present study, we categorized SB further by the developmental phases during which they were exhibited, into early (ages 0–6.99), mid- (ages 7–11.99), and late (ages 12–17.99) childhood. SB that were reported to persist across two or more of these developmental phases were categorized as persistent SB (early–mid, mid–late or early–late childhood).

#### Exposure to Sexual Abuse

Indications of sexual victimization prior to the index offense were collected from the case files and coded dichotomously into present/not present. Sexual victimization was defined as any sexual interaction with an adult person or any forced sexual activities by a peer. Kappa showed perfect agreement between raters (*κ* = 1.00).

#### Psychiatric Diagnoses

Psychiatric diagnoses at the time of the index offense were coded from the expert psychiatric and psychological reports in the case files according to the ICD-10 (World Health Organization, 1992) criteria. ICD-10 is the standard diagnostic classification system used in clinical settings in Switzerland. The following diagnostic categories were used: affective disorders (F30–F39), hyperkinetic disorders (F90), disruptive behavior disorders (F91, F92), neurotic, stress-related, and somatoform disorders (F40–F48), and substance related disorders (F10–F19). For the purposes of our study, an additional category of disorders related to sexual preference or development was included that combined disorders of sexual preference (F65), psychological and behavioral disorders associated with sexual development and orientation (F66), and sexual dysfunction not caused by organic disorder or disease (F52). Kappa indicated perfect agreement for the diagnostic categories (*κ* = 1.00).

#### Previous Offenses and Offense Characteristics

We coded information from the case files on previous sexual, nonsexual violent, and general offenses, including information on offenses for which no charges had been pressed. To counteract a possible overlap of SB reports with prior sexual offenses, only prior nonsexual violent offenses were examined. The following characteristics of the index offense were considered: child victim (i.e., at least three years younger than the JSO and younger than age 12), stranger victim (i.e., unknown or known to the JSO for less than 24 h prior to the index offense), multiple victims (at least two), and at least one male victim. Additionally, we categorized the severity of the index sexual offenses according to the offense-severity scale introduced by Aylwin et al. (2000). We further coded offenses with oral, vaginal, and/or anal sex, group offenses, and/or use of force as severe offenses.

#### Criminal Recidivism

Two sources were used to derive information on criminal recidivism. The first source was official data on offense types registered by the Swiss Federal Office of Justice and the Swiss Federal Statistical Office. Because Swiss juvenile justice authorities also supervise the course of court-ordered measures, there are separate juvenile justice case files including reports from supervisors, institutions, and other sources. Thus, as a second source, reports on criminal behavior from case files were also considered for coding criminal recidivism. Aside from a variable coding any criminal recidivism (any offence according to the Swiss criminal code; without juvenile justice administration offenses, e.g., breach of probation conditions), a variable coding sexual recidivism (crimes against the sexual integrity according the Swiss criminal code such as rape, indecent assault or sexual acts with children under the age of 16) and a variable coding nonsexual violent recidivism (according the Swiss penal code such as robbery, assault, acts of physical aggression) was distinguished. All JSO were followed for 365 days after conviction for the index offense. No time-at risk-measure was included in the analyses because secure inpatient or prison settings are extremely rare in Switzerland (Federal Statistical Office, 2021). Inter-rater agreement for the categories ranged from moderate to complete: general recidivism (*κ*_official_ = 1.00; *κ*_case files_ = 0.80), sexual recidivism (*κ*_official_ = 0.89; *κ*_case files_ = 0.79), and nonsexual violent recidivism (*κ*_official_ = 1.00; *κ*_case files_ = 0.60) (see also Barra et al., 2018).

#### J-SOAP-II

The revised Juvenile Sex Offender Assessment Protocol-II (J-SOAP-II; Prentky & Righthand, 2003) is a risk assessment tool for sexual and criminal recidivism among JSO ages 12–18. The J-SOAP-II total score is the sum of 28 risk item scores from four subscales: sexual drive/preoccupation items, impulsive/antisocial behavior items, intervention items, and community stability/adjustment items. The total score is interpreted relative to the maximum score. We used the German version of the J-SOAP-II (Schmelzle, 2004) in the present study (ICC J-SOAP-II total score = 0.74). The German version of the J-SOAP-II shows acceptable to excellent psychometric properties and evidence of predictive validity for sexual and general recidivism (Aebi et al., 2011; Barra et al., 2018; Quenzer & Dahle, 2010; Rettenberger et al., 2014).

### Statistical Analyses

Statistical analyses were performed with MPlus (Muthén & Muthén, 2017), IBM SPSS Version 26, and R Version 3.6.3 (R Core Team, 2020).

#### Receiver Operating Characteristics Analyses

Receiver operating characteristics analyses (ROC) analyses (Pintea & Moldovan, 2009) were performed to assess the predictive validity of the J-SOAP-II total score by the area under the curve (AUC) for criminal recidivism. Rice and Harris (2005) interpret AUC values analogously to Cohen’s effect size *d* as small (AUC = 0.556–0.639; *d* = 0.20–0.50), moderate (AUC = 0.639–0.714; *d* = 0.50–0.80), and large (AUC ≥ 0.714; *d* ≥ 0.80).

#### Identification of SB Subtypes

The presence of subtypes of JSO based on the eight dichotomous SB variables was investigated using latent class analysis (LCA). LCA assigns individuals to latent classes according to the individual pattern that they exhibit on a set of indicators. The entropy value indicates how well the latent classes can be differentiated (Masyn, 2013), and a value of at least 0.80 is recommended (Clark & Muthén, 2009). To decide which number of latent classes best fits the data, fit indicators such as the Akaike Information Criterion (AIC; Akaike, 1974), the Bayesian Information Criterion (BIC; Schwarz, 1978), and the sample-size adjusted Bayesian Information Criterion (aBIC; Sclove, 1987) are considered, and model solutions are tested against more parsimonious models with fewer classes using the Bootstrapped Likelihood Ratio Test (BLRT; McLachlan & Peel, 2000) and the Lo–Mendell–Rubin Likelihood Ratio Test (LMR LRT; Lo et al., 2001). Smaller fit indicators suggest a better model fit and parsimony, and model comparisons yielding significant results indicate a better model fit for a model with *k* latent classes compared to a model with *k*-1 latent classes. There are indications that the aBIC and the BLRT should be preferred to assess model fit and model comparisons respectively, in categorical LCA (Nylund et al., 2007). According to Nylund-Gibson and Choi (2018), it is common that fit indices do not clearly support just one solution but that there is support for multiple solutions. Importantly in LCA model selection, interpretability of the latent classes must be considered (Nylund et al., 2007). To counteract the likelihood function converging on a local instead of a global solution, resulting in the selection of a model with too many classes, 1000 random starts were implemented (Geiser, 2011; McLachlan & Peel, 2000; Uebersax, 2000).

#### Sexualized Behavior Group Comparisons

To compare subtypes of SB for psychopathology, victimization, and criminal characteristics (previous violent offenses, offense characteristics, and criminal recidivism), we used *χ*^2^- and Fisher’s tests for categorical variables and *t*-tests to examine differences between means or Mann–Whitney *U-*tests when requirements for parametric testing were not met. To counter type-I error inflation due to multiple comparisons, the Benjamini–Hochberg correction (Benjamini & Hochberg, 1995) was employed using the stats package in R (R Core Team, 2020). A posteriori power analyses for comparisons of the latent classes were performed using the pwr package in R (Champely, 2020).

## Results

### Descriptive Results

Table 1 shows the prevalence of the eight SB variables by the developmental period in which they were exhibited. Of the total sample, 93 (40.4%) JSO showed SB prior to the index offense. SB tended to be exhibited in mid- and late childhood, that is, in temporal proximity to the index sexual offense. Early SB exhibited prior to age 7 were seldomly reported. In about a quarter of cases with SB, the SB persisted from one developmental phase to the next, predominantly from mid- to late childhood, however, only in a few cases was this the same type of SB (Table 1). Where exposure to sexual abuse was recorded in the case files, the indications of sexual abuse preceded indications of SB in the majority of cases (98.7%). In two cases, the timing of the exposure to sexual abuse was unknown, and in one case, SB preceded sexual abuse according to case file information.

**Table 1:** Prevalence of sexualized behavior prior to the index offense

	Developmental period^a,b^						
	At any time prior to index offense	Early childhood (ages 0–6.99)	Mid-childhood (ages 7–11.99)	Latechildhood (ages 12–17.99)	Persistentearly to mid-childhood	Persistentmid- to latechildhood	Persistentearly to latechildhood
	*N *(%)	*N *(%)^a^	*N *(%)	*N *(%)	*N *(%)	*N *(%)	*N *(%)
Touching others inappropriately	43 (18.7)	1 (2.3)	11 (25.6)	26 (60.5)	1 (2.3)	4 (9.3)	0 (0.0)
Undressing/showing genitalia inappropriately	12 (5.2)	0 (0.0)	3 (25.0)	7 (58.3)	1 (8.3)	1 (8.3)	0 (0.0)
Touching genitalia/masturbation in the presence of others	8 (3.5)	0 (0.0)	3 (37.5)	3 (37.5)	0 (0.0)	2 (25.0)	0 (0.0)
Early masturbation^c^	24 (10.4)	1 (4.2)	22 (91.6)	–	1 (4.2)	–	–
Early exposure to pornography^c^	28 (12.2)	2 (7.1)	26 (92.9)	–	0 (0.0)	–	–
Excessive masturbation	13 (5.7)	1 (7.7)	2 (15.4)	10 (76.9)	0 (0.0)	0 (0.0)	0 (0.0)
Excessive pornography	19 (8.3)	0 (0.0)	1 (5.3)	18 (94.7)	0 (0.0)	0 (0.0)	0 (0.0)
Excessive sexual thoughts	17 (7.4)	1 (5.9)	2 (11.8)	11 (64.7)	0 (0.0)	2 (11.8)	1 (5.9)
Any SB^d^	93 (40.4)^e^	2 (2.2)	29 (31.9)	36 (39.6)	2 (2.2)	20 (21.5)	2 (2.2)

^a^The age categories are mutually exclusive

^b^The percentages provided for the age categories in the rows refer to the respective row total (i.e. at any time prior to the index offense)

^c^Prior to age 12

^d^Age categorization across all SB types

^e^*n* = 2 of the JSO presenting any SB presented both early and late SB but no known SB during mid-childhood. These are not included in the age categorization

### Predictive Validity of the J-SOAP-II

The J-SOAP-II total score significantly predicted sexual, nonsexual violent, and general recidivism in the current sample (sexual recidivism AUC = 0.661, *p* = .001, LL = 0.566, UL = 0.756, nonsexual violent recidivism AUC = 0.768, *p* < .001, LL = 0.691, UL = 0.846, general recidivism AUC = 0.751, *p* < .001, LL = 0.687, UL = 0.815). Omitting item 7 lead to similar results (sexual recidivism AUC = 0.664, *p* = .001, LL = 0.571, UL = 0.758; nonsexual violent recidivism AUC = 0.773, *p* < .001, LL = 0.696, UL = 0.850; general recidivism AUC = 0.755, *p* < .001, LL = 0.692, UL = 0.819).

### Latent Class Analysis of Sexualized Behavior Profiles

**Fig. 1:**
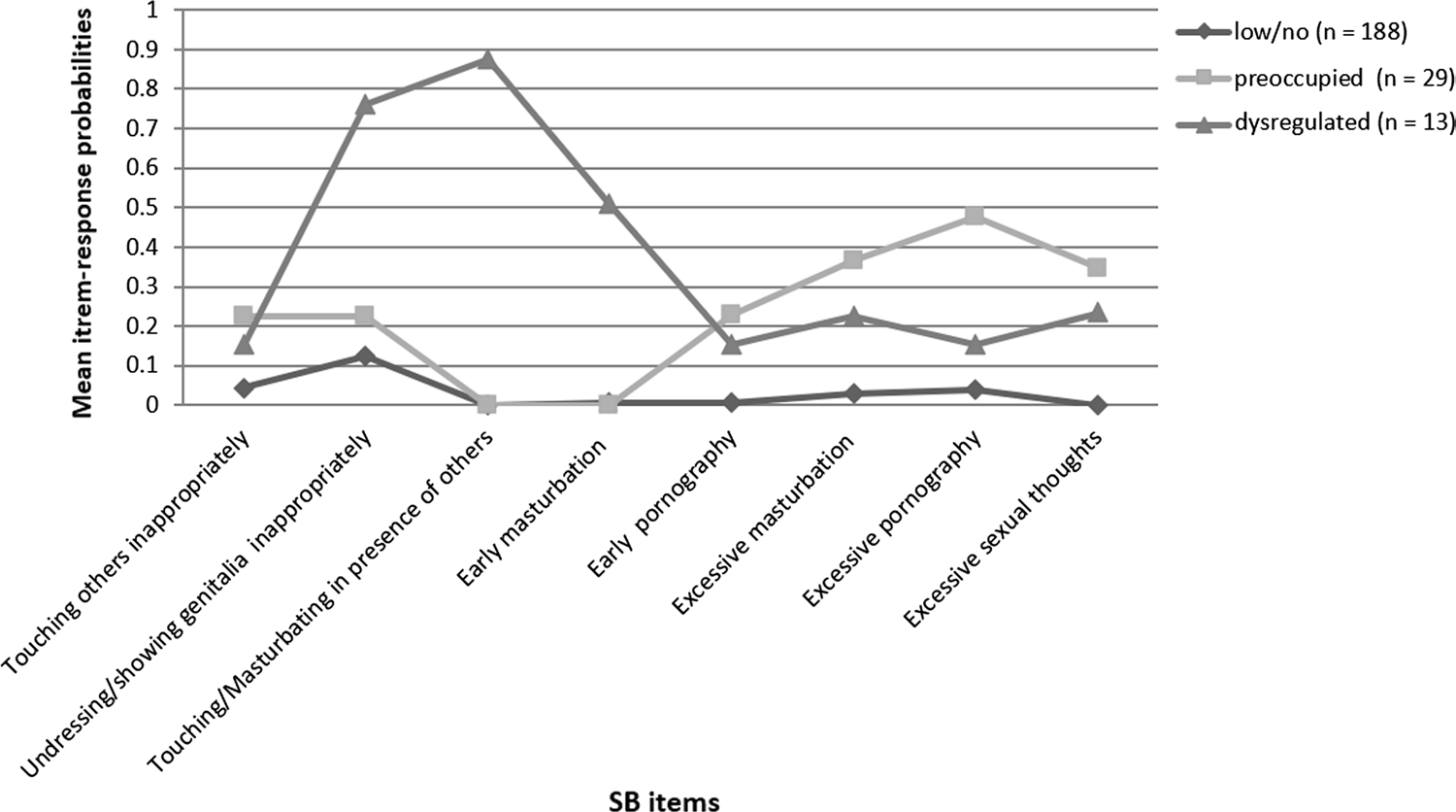
Three-class solution based on mean item response probabilities for eight sexualized behavior items

Table 2 shows the results of LCA for one to four classes. The two- and three-class solutions had similar statistical support. The BIC was smaller for the two-class model than for the three-class model; however, the AIC, aBIC, BLRT, and the overall entropy all supported the three-class model. Taking these criteria into consideration, and given previous literature from clinical and community samples of children suggesting that different subgroups exist among children with SB (e.g., Elkovitch et al., 2009; Fanniff et al., 2014), we concluded that the three-class solution represented the data best. Profiles of SB for the final three-class solution are shown in Fig. 1. The first class encompassed the majority of the sample (81.74%) and was characterized by low probabilities across all SB. We therefore labeled this class “low/no SB.” The second class encompassed 12.61% of the sample. This class showed the highest probabilities regarding excessive masturbation and excessive consumption of pornography. We coined this class “preoccupied SB.” A small percentage of the sample (5.65%) was assigned to the third class. This class showed the highest probability of exhibiting inappropriate behaviors directed toward others (i.e., inappropriately showing or touching/masturbating one’s own genitalia and touching others inappropriately), that is, crossing interpersonal boundaries. We therefore named this class “dysregulated SB.” A posteriori power analysis revealed insufficient power to detect small or medium effects between the dysregulated SB and preoccupied SB at a significance level of *p* < 0.05. Therefore, a combined SB group (*n* = 42) consisting of the preoccupied SB and the dysregulated SB was created for further analyses. Additional exploratory analyses for preoccupied SB and dysregulated SB were performed only when significant differences between low/no SB and combined SB were detected.

**Table 2:** Model parameters^a^ of latent classes of eight sexualized behavior items

Number of classes	Log likelihood	AIC	BIC	aBIC	*p* (VLM LRT)	*p* (LMR LRT)	*p* (BLRT)	Entropy
1	− 525.942	1067.89	1095.39	1070.03	–	–	–	–
2	− 485.319	1004.64	1063.09	1009.21	.001	.001	.000	.72
3	− 470.932	993.86	1083.26	1000.85	.048	.051	.000	.81
4	− 464.727	999.46	1119.79	1008.86	.331	.336	.667	.87

^*a*^*AIC* Akaike Information Criterion, *BIC* Bayesian Information Criterion, a*BIC* sample-size adjusted Bayesian Information Criterion, *VLM LRT* Vuong-Lo-Mendell-Rubin Likelihood Ratio Test, *LMR LRT* Lo-Mendell-Rubin Likelihood Ratio Test, *BLRT* Bootstrapped parametric Likelihood Ratio Test

### Group Comparisons

Table 3 shows the prevalence and group differences of demographic variables, the exposure to sexual abuse, and the presence of ICD-10 psychiatric disorder categories. Combined SB and low/no SB groups did not differ in age, low SES, foreign nationality, or exposure to sexual abuse. The combined SB had a significantly higher rate of Axis-I disorders than the low/no SB and more specifically disruptive behavior disorders. Further exploratory tests within the combined SB showed that there was no significant difference between the preoccupied SB and dysregulated SB in either the rate of any Axis-I disorders (Fisher’s exact test, *p* = 1.000), or disruptive behavior disorders (*χ*^2^ = 0.006(1), *p* = .936). Following the index sexual offense, 71.3% of the sample received some kind of group or individual psychotherapy. The combined SB group received therapy significantly more often than the low/no SB group.

**Table 3:** Group comparisons of demographic variables, exposure to sexual abuse, and psychiatric disorders

Variable	Complete sample (*N* = 230)	Sexualized behavior combined^a^ (*n* = 42)	Low/No sexualized behavior (*n* = 188)	Combined sexualized behavior vs. low/no sexualized behavior	Cohen’s *d*/*φ*
	*N *(%)	*n *(%)	*n *(%)		
Age at index offense	*M* = 14.46 (*SD* = 1.49)	*M* = 14.90 (*SD* = 1.69)	*M* = 14.37 (*SD* = 1.43)	*U* = 4653 *p* = .171	*d* = 0.338
Low SES	35 (15.2)	9 (21.4)	26 (13.8)	*χ*^2^ = 1.536 *p* = .338	ϕ = 0.082
Foreign nationality	73 (31.7)	14 (33.3)	60 (31.9)	*χ*^2^ = 0.032 *p* = .919	ϕ = 0.012
Exposure to sexual abuse	32 (13.9)	9 (21.4)	23 (12.2)	*χ*^2^ = 2.423 *p* = .264	ϕ = 0.103
Psychiatric disorders at time of index offense					
Disorder(s) related to sexual preference/ development	13 (5.7)	4 (9.5)	9 (4.8)	^b^*p* = .386	ϕ = 0.079
Affective disorders	21 (9.1)	7 (16.7)	14 (7.4)	^b^*p* = .168	ϕ = 0.124
Hyperkinetic disorder	65 (28.3)	17 (40.5)	48 (25.5)	χ^2^ = 3.782 *p* = .163	ϕ = 0.128
Disruptive behavior disorders	88 (38.3)	23 (54.8)	65 (34.6)	χ^2^ = 5.923 *p* = .049	ϕ = 0.160
Anxiety-related disorders	7 (3.0)	1 (2.4)	6 (3.2)	^b^*p* = .918	ϕ = -0.018
Substance-related disorders (abuse/dependency)	16 (7.0)	5 (11.9)	11 (5.9)	^b^*p* = .330	ϕ = 0.092
Any Axis-1 diagnosis	136 (59.1)	35 (83.3)	93 (49.5)	*χ*2 = 15.953 *p* = .006	ϕ = 0.263
Psychotherapy following the index offense	164 (71.3)	37 (88.1)	127 (67.6)	*χ*2 = 7.080 *p* = .036	ϕ = 0.175

^a^Both SB groups (dysregulated *n* = 13 and preoccupied *n* = 29) combined

^b^Fisher’s exact test

Table 4 shows the prevalence and group differences of previous violent offenses, characteristics of the index offense, J-SOAP-II total score, and criminal recidivism. The combined SB group showed a higher rate of nonsexual violent offenses prior to the index sexual offense compared to the low/no SB group. A further exploratory *χ*^2^-test within the combined SB showed no significant difference between preoccupied SB and dysregulated SB in the rates of prior nonsexual violent offenses (*χ*^2^ = 0.563(1), *p* = .453). No differences were found for the characteristics of the index offense between the low/no SB and the combined SB group.

**Table 4:** Group comparisons of previous violent offenses, characteristics of the index offense, J-SOAP-II total score, and criminal recidivism

Variable	Complete sample (*N* = 230)	Combined sexualized behavior (*n* = 42)	Low/No sexualized behavior (*N* = 188)	Combined sexualized behavior vs. low/no sexualized behavior	Effect size ϕ /Cohen’s *d*
	*N*(%)	*n*(%)	*n*(%)		
Nonsexual violent offenses prior to index offense	75 (32.6)	23 (54.8)	52 (27.7)	*χ*^2^ = 11.475 *p* = .006	ϕ = 0.223
Characteristics of the index offense^a^					
Severe offense^b^	145 (63.0)	30 (71.4)	115 (61.2)	*χ*^2^ = 1.551 *p* = .338	ϕ = 0.082
Stranger victim	29 (12.6)	8 (19.1)	21 (11.2)	*χ*^2^ = 1.933 *p* = .328	ϕ = 0.092
Male victim	67 (29.1)	12 (28.6)	55 (29.3)	*χ*^2^ = 0.008 *p* = .930	ϕ = -0.006
Multiple victims	50 (27.7)	7 (16.7)	43 (22.9)	*χ*^2^ = 0.777 *p* = .520	ϕ = -0.058
Child victim^c^	118 (51.3)	22 (52.4)	96 (51.1)	*χ*^2^ = 0.024 *p* = .919	ϕ = 0.010
J-SOAP-II sum score	*M* = 20.06 (*SD* = 9.99)	*M* = 27.31 (*SD* = 10.06)	*M* = 18.44 (*SD* = 9.26)	*U* = 5810 *p* = .006	ϕ = 0.917
J-SOAP-II sum score (excluding item 7, Sexual drive / Preoccupation)	*M* = 19.46 (*SD* = 9.70)	*M* = 25.88 (*SD* = 9.80)	*M* = 18.03 (*SD* = 9.10)	*U* = 5675.5 *p* = .006	ϕ = 0.830
Recidivism (within 365 days)					
Sexual	24 (10.4)	0 (0.0)	24 (12.8)	*p* = .038^d^	ϕ = -0.161
Nonsexual violent	40 (17.4)	9 (21.4)	31 (16.5)	*χ*^2^ = 0.583 *p* = .576	ϕ = 0.050
Any	89 (38.7)	17 (40.5)	72 (38.3)	*χ*^2^ = 0.069 *p* = .918	ϕ = 0.017

^a^Including attempts

^b^Oral, vaginal, or anal sex, group offense or use of force during the offense according to the offense-severity scale byAylwin et al. (2000)

^c^One or more victims younger than 12 years and age difference between victim and JSO more than three years

^d^Fisher’s exact test

The J-SOAP-II total score of combined SB was significantly higher than that for low/no SB. This was also the case when J-SOAP-II item 7, sexual drive/preoccupation, was excluded from the J-SOAP-II total score. A further exploratory examination within combined SB showed that preoccupied SB and dysregulated SB did not differ significantly in the J-SOAP-II total score (*U* = 182.0, *p* = .872), also when item 7 was removed from the J-SOAP-II total score (*U* = 182.5, *p* = .872). Combined SB showed a significantly lower rate of sexual reoffending than low/no SB. None of the JSO of the combined SB group reoffended sexually within one year. In contrast, 12.8% of the low/no SB group reoffended sexually in the same time frame. This finding was statistically significant. No significant differences emerged between low/no SB and combined SB regarding nonsexual violent and general recidivism.

## Discussion

The present study aimed to identify patterns of SB among JSO and to analyze their relationships with demographic variables, exposure to sexual abuse, psychiatric disorders, offense characteristics, and sexual recidivism. To the best of our knowledge, no previous study has examined patterns of SB in terms of excessive sexual drive and preoccupation and independently of sexual deviance and offending among JSO. Improved knowledge about the characteristics of SB and their relations to psychopathology and future criminality may inform risk assessment and suggest actionable targets for treatment interventions of JSO.

Notably, despite only including JSO for whom a forensic expert assessment was available, the sample appears representative regarding the proportion of foreign nationality among JSO in Switzerland (Barra et al., 2021). However, the current sample showed higher mean J-SOAP-II total scores (20.0 vs. 14.9) and higher rates of sexual, non-sexual violent and general recidivism compared to a population sample of Swiss JSO (Barra et al., 2018). Nevertheless, is not an extreme sample regarding risk and recidivism in the German-speaking countries. Risk and recidivism data are comparable to those of a German sample of 80 minors under suspicion of having committed a sexual offense (Rettenberger et al., 2014).

### Heterogeneity of Sexualized Behavior

Three subgroups of JSO were distinguished with regard to their SB profiles: a group with low or no sexualized behavior, a sexually preoccupied group, and a sexually dysregulated group. The majority of JSO included in this study had no known history of SB prior to their first sexual offense. This appears to be in line with previous research by Dennison and Leclerc (2011), who found 81.6% of their sample of JSO did not show inappropriate sexual behavior inappropriate sexual behavior, defined there as exhibitionism, voyeurism, obscene phone calls, using deviant pornography, and/or using erotic hotlines, although this definition only partly overlaps with the definition of SB used in the present study. Furthermore, this result is in agreement with previous studies that suggest several risk factors for sexual offending that are not related to non-normative sexual development, including social skills deficits, general antisocial attitudes, or negative peer interactions (Aebi et al., 2012; McCann & Lussier, 2008; Worling & Långström, 2006). It appears that few JSO commit sexual offenses predominantly due to insufficient ability to control sexual urges and impulses.

In addition to the large group with low/no SB, two smaller subtypes with distinct patterns of SB were identified: (1) a preoccupied SB subtype, characterized by early or excessive preoccupation with sexuality in the form of sexual thoughts, masturbation, and pornography, and (2) a dysregulated SB subtype, characterized by inappropriate sexual behaviors in the presence of or directed toward others. To our knowledge, no research has been conducted on subtypes of JSO with regard to SB specifically.

Spearson Goulet and Tardif (2018) used cluster analysis to examine differences among JSO on a number of dimensions of sexuality (atypical and normative fantasies and experiences, drive, body image, pornography, first masturbation, onset of sexual interest, and first exposure to sex). They identified a largely normative, a sexually restricted (less interested/invested in sexuality) and an overinvested subgroup of JSO, suggesting substantial heterogeneity among JSO regarding the domain of sexuality and sexual development more generally. Research from clinical/community samples does not offer an established typology of SB among children or adolescents either (Chaffin et al., 2008; Fanniff et al., 2014). However, the low/no SB, dysregulated SB, and preoccupied SB subtypes suggested by the current study do appear to be consistent with a common categorization in clinical settings of youth with SB into children with predominantly interpersonal SB (i.e., SB "involving another individual"; Allen, 2017, p. 192; DeLago et al., 2020) and children with self-focused SB (Allen, 2017; Elkovitch et al., 2009). Children with interpersonal SB appear to exhibit more developmental difficulties such as skills deficits and higher rates of abuse experiences than typically developed youth (Elkovitch et al., 2009).

The current findings point to the importance of preoccupied and dysregulated SB in a small subgroup of JSO. SB may lower the threshold to commit sexually abusive behaviors in some youth. The current findings may encourage further specific research to understand the processes of sexual inhibition and activation (Janssen & Bancroft, 2007) also in the context of juvenile sexual offending.

### Association of Sexualized Behavior with Indications of Exposure to Sexual Abuse and Psychiatric Disorders

For 12.2% of JSO in the low/no SB group and 21.4% in the combined SB group, there was an indication of exposure to childhood sexual abuse. While this difference was not significant, the direction of the difference is in line with most previous research with JSO, clinical, and child protection samples, suggesting an association between exposure to sexual abuse and SB (e.g., Allen, 2017; Davis & Knight, 2019; Letourneau et al., 2004; Tarren-Sweeney, 2008; Wamser-Nanney & Campbell, 2019). However, in line with previous research, indications of exposure to sexual abuse were present only in a minority of children with SB (minority of the combined SB group) (e.g., Allen, 2017; Silovsky & Niec, 2002). The current result, thus, ties in with previous literature suggesting that other factors besides exposure to sexual abuse, such as other types of adverse childhood experiences, play a role in the etiology of SB (Elkovitch et al., 2009).

As indicated by previous research (Seto & Lalumiere, 2010), we found a high prevalence of mental health problems in the sample. However, there was a difference between the SB groups: The combined SB group showed very high rates of psychiatric disorders, with more than 80% of JSO in this group meeting the criteria of an ICD-10 psychiatric disorder. In contrast, low/no SB had a significantly lower prevalence rate of 52.7% for any psychiatric disorder, although the effect size remained small. Similarly, Letourneau et al. (2008) found that youths with sexual behavior problems had a higher rate of psychopathology than youths without sexual behavior problems in a sample of adolescents referred to outpatient treatment for behavior problems, with the former scoring higher on internalizing and externalizing problems. Data from child protection samples also suggest that children with SB present higher rates of mental health problems than children without SB (e.g., Baker et al., 2008; Szanto et al., 2012). It is likely due to this high burden of psychopathology that JSO in the combined SB group of the current study received psychotherapy at a significantly higher rate than low/no SB JSO.

In the present study, the combined SB group presented a significantly higher rate of disruptive behavior disorders than low/no SB. The notion of a partial overlap in the etiology of externalizing problems and SB has been supported by studies across clinical, child protection, and community samples of children (e.g., Allen, 2017; Ensink et al., 2018; Lévesque et al., 2010; Lussier & Healey, 2010; Malvaso et al., 2020; Meyer-Bahlburg et al., 2000; van Goozen et al., 2002; Wamser-Nanney & Campbell, 2019), thus suggesting that dysregulation, that is, deficits in the regulation of emotions, behavioral impulsivity, and attention, may be common factors for a range of social behavior problems, including sexual behaviors (Aebi et al., 2020; Aitken et al., 2019). Further, neuropsychological deficits such as impaired cognitive and emotional functioning affecting self-regulation discussed in the etiology of antisocial and disruptive behavior (van Goozen et al., 2022) may tie in SB, disruptive behavior and criminal behavior.

### Association of Sexualized Behavior with Previous Violent Offenses, Characteristics of the Index Offense, J-SOAP-II Total Score, and Criminal Recidivism

In the present study, the combined SB group was found to have significantly higher rates of prior nonsexual violent offenses. This finding supports the suggestion that general impulse control deficits or antisocial attitudes may become of relevance for SB among JSO. Although we did not find a specific association of SB with ADHD in our study, previous research has suggested that limited impulse-control in childhood and adolescence may present a major risk factor for committing violent offenses (see meta-analysis by Mohr-Jensen & Steinhausen, 2016). The present nonsignificant results regarding offense characteristics appear in line with previous findings by Spearson Goulet and Tardif (2018) suggesting that JSO with exacerbated sexuality do not differ from JSO with a normative pattern of sexuality with regard to victim characteristics (e.g., multiple victims, male victim). The assumption that JSO with SB would present a more severe picture regarding the offense characteristics (e.g., with regard to the severity of the index offense) is not supported in the current data. SB as defined in the present study should not be confounded with sexual deviance and seem not to be associated with the severity of sexual crimes among JSO.

As expected, JSO in the combined SB group were found to score significantly higher on the J-SOAP-II than the low/no SB. This was not due to the specific item measuring sexual behavior problems of the J-SOAP-II (item 7), because the large effect was retained when this item was omitted from the score. The higher sum scores of the J-SOAP-II thus appear to arise from other risk factors possibly associated with SB (Vitacco et al., 2009). Overall, in the current sample the J-SOAP-II predictive validity for sexual recidivism was moderate.

We found no significant difference between the two groups in nonsexual and general recidivism rates, which is in line with a previous study by Letourneau et al. (2008). Those authors further report no significant difference in sexual recidivism between youth with and without sexual behavior problems (sexual recidivism rate < 2% in both groups). In the present study, the combined SB group showed a significantly lower rate of sexual recidivism (i.e., none) than low/no SB (sexual recidivism rate: 12.8%). Although the effect size was small and caution is warranted due to the small sample size and the limited time range for recidivism, the current findings may indicate that an intense focus on SB is not warranted for preventing further sexual offenses of JSO. Although we do not fully understand this result, some possible explanations merit note: First, this finding may reflect the dynamic nature of SB relating to sexual drive/preoccupation across childhood. In childhood and early adolescence, sexual behavior and sexual interests undergo substantial development with rapid changes and are influenced by contextual factors (e.g., Smith et al., 2005; Wurtele & Kenny, 2011). Thus, inappropriate and excessive sexual behaviors and preoccupations are often transient (Carpentier et al., 2006; Lussier et al., 2018; Steinberg, 2010). Secondly, social and cognitive maturation und social learning processes during adolescence lead to improved sexual control and to better handling of sexual feelings and impulses. Youth learn to cope better with sexual urges, and improved knowledge of sexual laws and fear of further consequences may also result in changes in sexual behaviors. Thirdly, SB may have responded well to intervention delivered from forensic service providers and mental health specialists, as previous research suggests (Carpentier et al., 2006; Letourneau et al., 2008). However, the current data did not provide details of the kind of treatment service provided to JSO with SB.

The high rates of nonsexual recidivism of JSO in the present sample are in line with previous research (Caldwell, 2016). Given the risk for general and violent recidivism, a disproportionate focus on SB does not appear justified. Rather, responses to juveniles (sexual) offending should follow the general principle of the Risk-Need-Responsivity (RNR) model toward individualized assessment and treatment of youth fostering rehabilitation and reintegration (Brogan et al., 2015; Hoge, 2016; Ter Beek et al., 2018). While the different rates of psychotherapy may be a consequence of more psychopathology in the SB group, as previously noted, it may also have resulted from an inadequate focus of clinicians on “sexual” issues rather than on conduct disorders and general antisocial attitudes.

### Strengths and Limitations

The present study has several strengths, such as the use of a population sample representing the majority of juveniles who were convicted of a sexual offense in the German-speaking part of Switzerland between 2007 and 2014 and the use of case file information to identify recidivism in addition to officially registered recidivism. Moreover, eight SB related to managing sexual urges and impulses were assessed independently of sexual deviancy and sexual offending.

However, a number of limitations merit note. First, all variables used were coded solely from case file information and official databases. Since the case files investigated had been created by the juvenile justice system for internal use, some information relevant to variables in this study may have been limited. Some variables were coded from reports by the same psychological and psychiatric experts, which was not statistically controlled for. The heterogeneity and the nature of expert opinions included in case files may further limit the current findings. No formal reliability estimates were available for psychiatric disorders assessed by clinicians that were coded directly from files. Due to the selection of only those JSO from the sample for whom expert reports were available, the generalizability of the current results may be limited. Moreover, the selection criteria substantially reduced the sample size, and despite the combination of two of the classes, dysregulated SB and preoccupied SB, power remained insufficient to detect small effects. No specific information on treatment was coded from the case files. Finally, despite the inclusion of nonofficial recidivism reported in the case files, some other recidivism will still have gone unreported.

### Conclusions

Research into JSO predominantly investigates atypical sexual development and/or behavior in terms of deviant sexual fantasies, interests, arousal, and behaviors (e.g., sexual interest in preadolescent children, violent sexual fantasies etc.), or considers a combination of SB and deviant sexuality either of which found support as a risk factor for sexual recidivism among JSO (e.g., Clift et al., 2009; Dennison & Leclerc, 2011; Kenny et al., 2001; McCann & Lussier, 2008; Worling & Långström, 2006). As a main difference, the present study operationalized SB exclusively in terms of early and excessive sexual behaviors and preoccupations not in terms of sexual deviance. The results of the present study are thus not necessarily in contrast to previous research but expand the existing knowledge of JSO. While sexual deviance has found support as a risk factor for sexual recidivism among JSO, the current results suggest that this may not be the case for SB relating to sexual drive and preoccupation. Consequently, conflating a wide range of aspects of sexual drive and preoccupation with sexual deviance as a single risk factor—as is currently the case in the J-SOAP-II—appears questionable. Future research should differentiate sexual drive, preoccupation, and impulsivity from sexual deviance in JSO and address sexual excitation and sexual inhibition processes (Janssen & Bancroft, 2007) and executive functions in those JSO with SB and/or impulsive behaviors. Given the heterogeneity of JSO, an in-depth analysis of SB in different subtypes of JSO appears necessary (e.g., by victim age preference; Fix et al., 2019; Prentky et al., 2000). However, research on JSO should not overemphasize the role of sexuality in JSO and should consider criminogenic needs more generally.

Several treatment programs for compulsive sexual behaviors and pornography addiction exist for adults (Efrati & Gola, 2018). However, these approaches seem not to be appropriate for JSO with SB, in light of the high rate of associated mental health problems. Problematic sexual behaviors in youth should be understood in the context of complex biological and social developmental processes. Forensic mental health providers should carefully assess SB in the broader context of externalizing behaviors in JSO. In line with the Risk-Need-Responsivity (RNR) principles (Andrews & Bonta, 2010), forensic mental health service providers should focus on the broad span of individual criminogenic needs to tailor interventions for JSO.
